# The urgent need for biosecurity education within the agribusiness sector

**DOI:** 10.3389/fmicb.2026.1735642

**Published:** 2026-02-11

**Authors:** Olivia Ibbotson, Lijun Shang

**Affiliations:** 1 School of Human Sciences, London Metropolitan University, London, United Kingdom; 2 Biological Security Research Centre, London Metropolitan University, London, United Kingdom

**Keywords:** agribusiness sector, biosecurity, BTWC, curriculum, education

## Abstract

Biosecurity within the agribusiness sector is an often-neglected and forgotten area. Biosecurity vulnerabilities within the sector hold significant global risks, which are emphasised by the ever-growing threat of climate change, the presence of global conflicts, and the advancements made in science and technology. Biosecurity vulnerabilities also hold a multitude of public health (physical and psychological), environmental, economic, and political consequences. In this study, we systematically surveyed 199 university-taught courses relating to agribusiness to determine the extent to which biosecurity risks were addressed. We found that only 4% of surveyed courses contained clear biosecurity elements, thus exhibiting a huge educational gap. Among these 4%, zero courses addressed agroterrorism, agro-crime, or bioweapons, including the economic, physical, psychological, and political implications. We therefore argue for the development of an internationally coordinated curriculum that embeds biosecurity concepts into established courses. This will hugely enhance existing topics taught in courses to meet the urgent need in new circumstances derived from new science and technology.

## 1 Introduction

Biosecurity in the agribusiness sector is an often-neglected and forgotten area of biological and chemical security. Vulnerabilities within the agribusiness sector hold significant global risks to the population, and the ever-growing threat of climate change, the increasing presence of national/international conflicts and unstable geo-political environments, in addition to advancements made in science and technology, draw light to the serious risks that have and are arising to the agribusiness sector (Ibbotson and Shang, 2025). Rapid advancements in science and technology hold significant advantages; however, they also pose great risks, and as such, advancements have allowed technological/biochemical manipulations to be easier than ever before. These advancements lead to question on the presence of Dual-Use Research of Concern and Dual-Use technology in agribusiness. Moreover, the specific biosecurity vulnerabilities that pose a threat to the agribusiness sector can be exploited for harm by the minority, resulting in various consequences to public health (physical and psychological) and the environment, causing disarray to a state’s socio-economic stability, in addition to the harm a state’s Gross Domestic Product (GDP) may suffer.

The term Agribusiness, as defined by David and Goldberg (1957), is “the complete number of all operations involved in the cultivation, production, farm operations, processing, and distribution of agricultural commodities and goods derived from them” (Davis, 1957). Biosecurity, as defined under the Biological and Toxin Weapons Convention (BTWC), is the “protection, control, and accountability measures implemented to prevent the loss, theft, misuse, diversion, or intentional release of biological agents and toxins, and related resources as well as unauthorised access to, retention, or transfer of such materials” (EU Working Paper, 2022).

When discussing the specific biosecurity risks/vulnerabilities to the agribusiness sector, it is useful to subcategorise into animals, food, and plant/environmental biosecurity. It is also important to assess how these subcategories interlink and how attacks on one area of agribusiness may produce a ‘domino effect’ on the entire supply chain. For example, food biosecurity is dependent on the whole food supply chain, encompassing the entire agribusiness sector. Biosecurity in the agribusiness sector relates to the prevention, detection, and management of intentional and unintentional contaminations/attacks. An intentional attack in the agribusiness sector using biological/chemical weapons would be classified as agroterrorism, which is a subset of bioterrorism. Agroterrorism specifically targets livestock and crop production. If, however, the deliberate acts are motivated by financial or personal gain, they may be classed as an agro-crime. The specific act of protecting food from intentional threats is classed food defence. Food defence, as defined by the Global Food Safety Initiative, is the “process to ensure security of food and drink, food ingredients, feed, or food packaging from all forms of intentional malicious attack, including ideologically motivated attacks leading to contamination or unsafe product” (Spink, 2023).

Due to the disastrous consequences that arise when biosecurity is neglected within the industry or in the event of an unintentional/intentional shock event, it is of great importance to educate future experts. Awareness raising and education of biosecurity within the Agribusiness sector is equally important as any other biosecurity area.

This study aims to examine the gap in biosecurity education in the agribusiness sector, with a focus on university-taught modules at Master’s and Undergraduate levels. University-taught modules were surveyed at a range of European Universities, focusing on those with research interests relating to agriculture, environment/plants, and food safety/security. The study will delve into biosecurity education in the agribusiness sector, before finally proposing how the biosecurity educational gap may be addressed through proactive approaches and the impact these may pose.

We start to look at the education within the agribusiness sector via surveying university-taught modules (Master’s and Undergraduate) to determine the extent to which biosecurity risks were addressed. Biosecurity risks, as discussed above, can be classed as unintentional or intentional and may result in a shock event. Risks may be established as past and present risks, or those that may be a future biosecurity concern. The biosecurity element of modules was analysed to determine what topics were addressed, such as agroterrorism, bioweapons [Biological and Toxin Weapons Convention (BTWC) + Chemical Weapons Convention (CWC)], epidemiology, intentional/unintentional contamination, pests, and zoonotic diseases.

We therefore argue for the development of an internationally coordinated curriculum that embeds biosecurity concepts into established courses. This will hugely enhance existing topics taught in courses to meet the urgent need in new circumstances derived from new science and technology.

## 2 Methodology

Biosecurity education within the agribusiness sector was analysed via surveying university-taught modules at Masters and Undergraduate levels. Universities were located within the European Union and Economic Area in addition to England and Scotland. Universities were selected due to their research interests focused on or in relation to agriculture, environmental/plant sciences, and food safety/security. EduRank.org was used to assist in the selection of universities, using the parameters “biology” and “agricultural sciences.” The Universities were ranked according to their research performance in the Agricultural Sciences. Course/module content and programmes were reviewed alongside course aims, in addition to future career prospects. As of September 2024, 199 courses have been surveyed from 60 Universities across 32 countries.

Data were collected via website analysis of secondary sources using the inclusion criteria: Agriculture, Agronomy, Animal, Biodiversity, Biology, Bio-resources, Biotechnology, Crops, Ecology, Environment, Food, Parasite, Pests, Plants, Poultry, Swine, Veterinary, and Wildlife.

Courses were analysed to determine if the following Biosecurity elements were addressed: agroterrorism, bioweapons (BTWC + CWC), epidemiology, intentional/unintentional contamination, pests, and zoonotic diseases. Courses were also analysed to determine if they address the societal (physical and psychological), environmental, economic, political, consequences, or global impacts of a shock event to the sector. After website scraping was completed via inclusion criteria, courses were analysed and differentiated into three categories: no biosecurity elements, some biosecurity elements, and clear biosecurity elements.

This methodology was based on what we could get from the website and what was the purpose we had for the survey. The further discussion of this methodology is provided below.

## 3 Results

**Figure 1:**
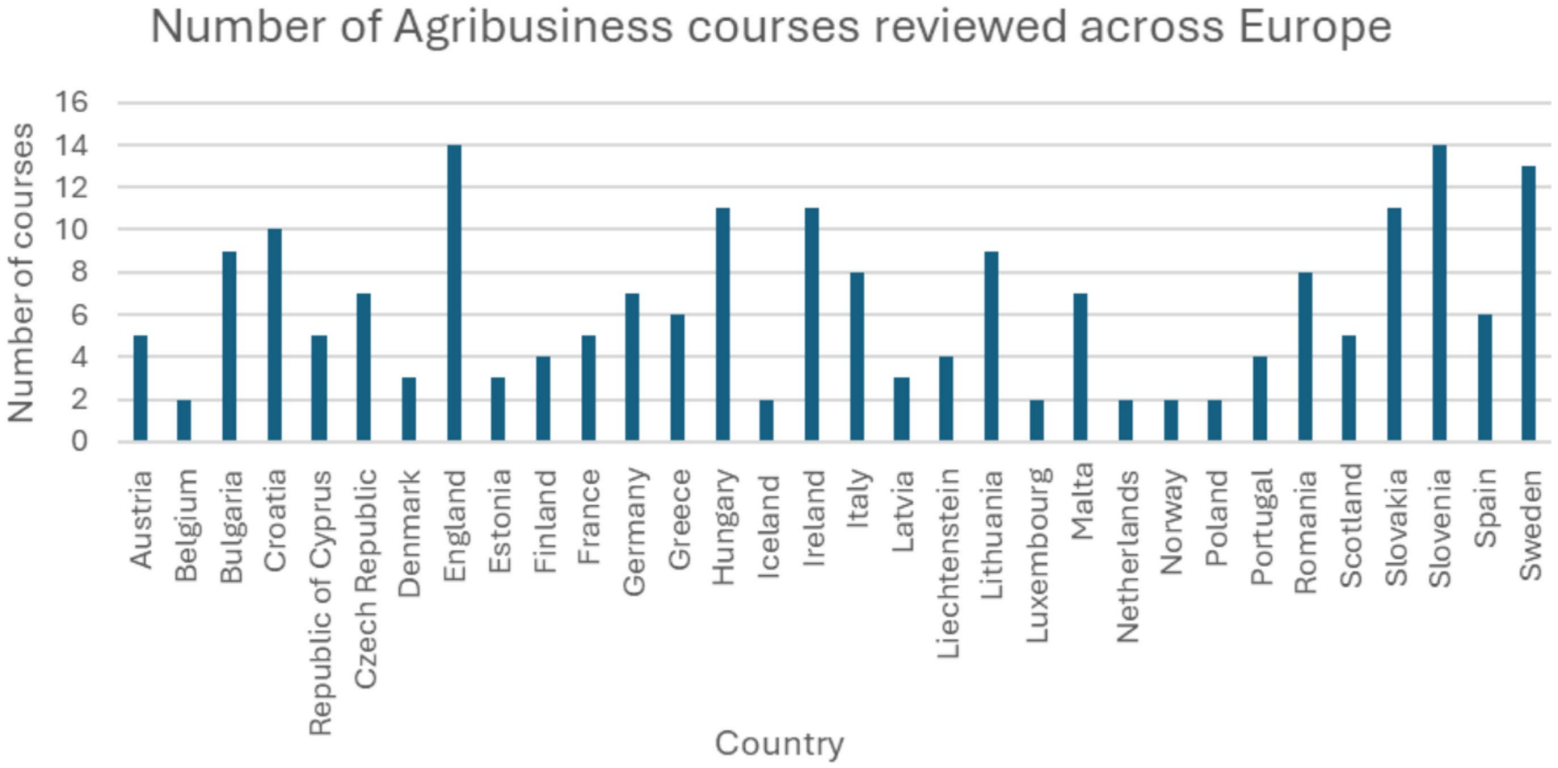
The geographical distribution of surveyed courses. This figure showed the 199 courses randomly distributed across the 32 countries within the European Union, European Economic Area, in addition to England and Scotland.

**Figure 2:**
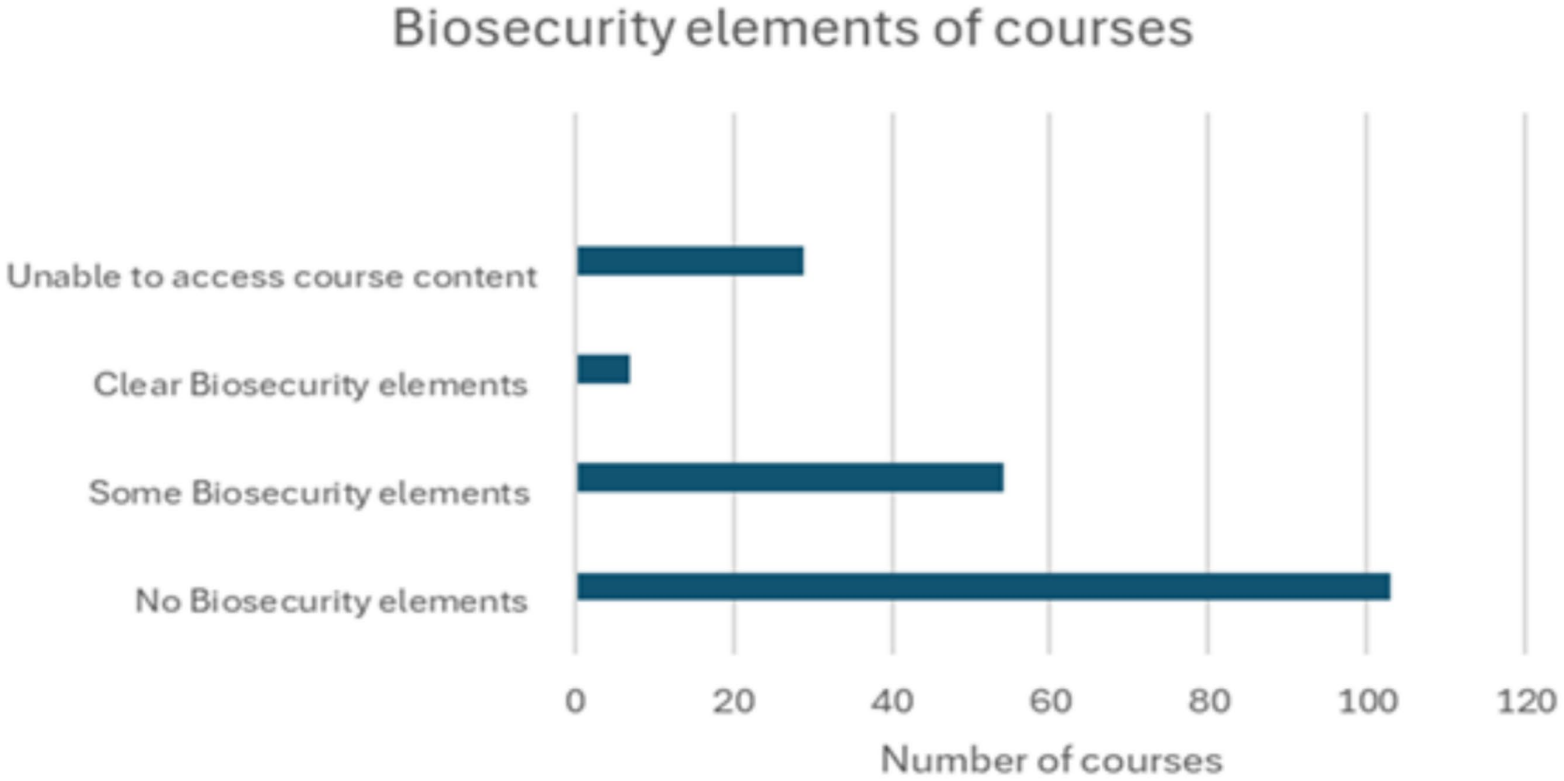
The biosecurity element distribution among the surveyed courses. Among the 199 surveyed courses, 29 courses had restrictions accessing course content, 7 courses contained clear biosecurity elements, 59 courses contained some biosecurity elements, and 103 courses contained no biosecurity elements.

In total, 199 university courses were surveyed, and a huge biosecurity educational gap was demonstrated. Figure 1 presents a distribution of the 199 courses surveyed from 32 countries across the European Union, European Economic Area, in addition to England and Scotland. It revealed that 52% of the courses contain no visible biosecurity elements, 27% revealed some biosecurity elements, 15% presented challenges accessing the course contents, and only 4% revealed clear biosecurity elements, as further illustrated in Figure 2 and Table 1. The dramatically low percentage of courses exhibiting clear biosecurity elements emphasises the need for biosecurity education within university courses relating to the agribusiness sector.

**Table 1:** The course categories as percentages regarding the biosecurity element.

Category	Number of courses	Percentage
No biosecurity elements	103	52%
Some biosecurity elements	54	27%
Clear biosecurity elements	7	4%
Unable to access course content	29	15%

Percentages of each course category exhibit the appearance of a huge biosecurity educational gap within university courses relating to Agribusiness, with only 4% of the course has clear biosecurity elements.

The survey also showed that all courses containing biosecurity elements were of a Master’s level, thus exhibiting the need for biosecurity elements to be implemented within Undergraduate courses. Moreover, identified courses were only from 5 countries, placing further emphasis on internationally coordinated biosecurity education, which is equally spread among countries.

Each course is identified to contain Clear Biosecurity elements, addressed biosecurity related topics such as epidemiology and pest control. It is not known to what extent advanced science and technology are discussed.

Of the 32 countries surveyed for this research, only Liechtenstein and Luxembourg did not reveal any courses visibly relating to agribusiness. As discussed in Table 2, the lack of visible courses does not mean that they are absent entirely, but just that they were not visible via the methodology used for this research.

**Table 2:** Countries did not contain visibly relevant courses.

Country	University
Liechtenstein	University of Liechtenstein
Liechtenstein	International Academy for Philosophy
Liechtenstein	Liechtenstein Institute
Liechtenstein	Private University in the Principality of Liechtenstein
Luxembourg	University of Luxembourg
Luxembourg	Sacred Hearts University

The unbalanced spread of Agribusiness education across European countries was identified as 6 universities from 2 countries did not visibly provide courses related to Agribusiness. An explanation for the lack of Agribusiness courses in Liechtenstein and Luxembourg may be that agricultural research in these countries appears to take place at a PhD level and seems to be focused on at separate research centers (outside of universities). However, the appearance of a lack of Agribusiness courses does not mean that they are absent entirely, just that they were not within the visible scope of this study, and the methodology used was unable to source them.

Among 7 courses from 5 countries that explicitly address biosecurity (Table 3), each course addresses topics such as epidemiology, accidental contamination, pests, and zoonotic diseases. Courses address the environmental impacts that may occur if biosecurity is neglected; however, only the University of Agronomic Sciences and Veterinary Medicine of Bucharest (Food Safety and Biosecurity) and Harper-Adams University address any economic impact.

**Table 3:** List of courses containing clear biosecurity elements.

Country, university, and course name	Biosecurity elements	Course details
Croatia University of Zagreb Farm Biosecurity	Covers farm animal housing hygiene; Air, soil, and water hygiene; Biosecurity standards and animal welfare; Sanitary parasitology; Sanitary microbiology; Clinical toxicology of insecticides and rodenticides; Immunisation as a biosecurity measure; Applied disinfection, disinsection, and deratisation; Applied veterinary epidemiology in farm animals; Farm biosecurity assessment	Masters Aimed at Doctors of Veterinary Medicine Students receive a specialist qualification for competency to work in the field of Farm Biosecurity Available via E-learning
Croatia University of Zagreb Agri-Food Chain Microbiology	Covers the impact of measures and inventions at pre-harvest and post-harvest levels: feed safety and feeding modulations; Hygiene and housing of farm animals; Farm biosecurity; Epizootiology/epidemiology of current biological hazards transmitted through the food chain, slaughterhouse hygiene and food processing hygiene; Impact of animal feeding on the safety of food of animal origin, Animal welfare and meat safety; Antimicrobial resistance in the food chain; Integrated meat safety system; Food microorganisms; Food preservation; Microbial spoilage of food; Beneficial food microbial spoilage of food; Alimentary infections and intoxications; Detection of microorganisms in the food chain	Masters Aimed at Doctors of Veterinary Medicine Practical training: Farms, slaughterhouses. Molecular, microbiological laboratories, and computer classrooms Most lectures and seminars are delivered via Distance learning Available via E-learning
England Harper-Adams University Plant Health and Biosecurity	Covers the control of plant pests and pathogens, plant pathology and entomology, reducing the introduction of invasive pests and pathogens, and minimising their impact if introduced. This course emphasises that biosecurity risks have increased with the globalisation of trade and travel. This course also covers the social, economic, and environmental impacts of biosecurity risks	Available as a PgC/ PgD/MSc Aimed at UK government staff with biosecurity responsibilities Aimed at those working with the plant businesses (plant importers, growers, and distributors) Master’s is delivered part-time over 2 years Aligned with competencies of the Royal Society of Biology Plant Health Professional Register
Italy University of Bologna Food safety and food risk management	Covers advanced and predictive food microbiology; Advanced food processing and packaging; Farm biosecurity and zoonotic diseases prevention; Foodborne risk traceability; evaluation of adverse health effects from human exposure to foodborne hazards; Innovative approach for risk assessment in microbiome food value chain	Masters Taught in English Delivered over 2 years Open access with assessment of personal competencies
Malta Malta College of Arts, Science, and Technology Veterinary Medicine	Covers diagnosis and treatment of medical and surgical conditions; Veterinary Public Health; Pharmacology; Animal health, welfare, and performance; Legal requirements; Animal anatomy, physiology, pathology, and ethology; Solutions to problems impacting One Welfare and respond effectively to various situations such as infectious diseases, biosecurity, and population control	MCAST -UAB Master of Science in Veterinary Medicine Delivered over 5 years Taught in English Blended learning
Romania University of Agronomic sciences and Veterinary Medicine of Bucharest Food biosafety	Covers Public Health and Food safety; Food quality and safety assurance by controlling the automatisation of technological flows; Advanced chemical, microbiological and toxicological procedures of food control and analysis; Additional products used in food industry processing; Economic strategies in food safety; Legislation in food safety and security; Biosecurity in the milk supply chain, meat supply chain, beekeeping supply chain, aquatic organisms supply chain and game supply chain	Masters Taught in Romanian Delivered over 2 years
Romania University of Agronomic sciences and Veterinary Medicine of Bucharest Food Safety and Biosecurity	Covers Public Health and food safety; Advanced chemical, microbiological and toxicological control and analysis of food; Economic strategies in food safety; Food safety policy and global food system; Additional products used in technological processes in the food industry; Biosecurity of producing raw materials of vegetable origin; Biosecurity of producing raw materials of animal origin; Risk assessment for food; Biorisk analysis in food	Masters Taught in English Delivered over 2 years

Seven courses from five countries, which contain clear biosecurity elements, were identified that explicitly address biosecurity.

It is very important to note, however, that zero courses were identified that address agroterrorism, bioweapons (including discussion on the BTWC and CWC), or intentional contamination such as agro-crime. Moreover, zero courses were identified that address the societal (physical and psychological) and political consequences of a Shock event.

Overall, the results reiterated a huge biosecurity educational gap within agribusiness-related courses at universities. The results suggested that this educational gap is international within Europe, and specifically, there is an absence of biosecurity within undergraduate courses. Moreover, courses do not visibly discuss or discuss in-depth the economic, physical, psychological, or political consequences of a biosecurity shock event.

The full survey of courses in this study is attached as Supplementary material.

## 4 Discussion

This survey has reviewed 199 courses from 60 Universities across 32 countries. Countries discussed span across the European Union, European-Economic area, in addition to England and Scotland. Universities were selected due to their research interests and number of publications on or in relation to Agriculture, Environmental/Plants, and Food Safety/Security. EduRank.org was used to assist in university selection.

As discussed above, biosecurity is of increasing importance within the agribusiness sector due to the growing threat of climate change, increasing populations, and the rise in food demand, the presence of various global conflicts, in addition to advancements made in science and technology.

Moreover, this emphasis or need for focus on biosecurity is further evidenced by only 7 out of the 199 (4%) courses reviewed containing clear biosecurity elements. The 7 identified courses explicitly addressed biosecurity, whether that be in the course name or module contents. Each course addressed key topics such as epidemiology, accidental contamination, pests, and zoonotic diseases. The biosecurity courses were also expected to address the socio-economic consequences that occur during or post-shock event. All 7 courses addressed environmental impacts but failed to address societal consequences. In total, 2 out of 7 courses addressed and discussed economic consequences; however, zero courses were identified that addressed the physical, psychological, and political consequences of a shock event. Furthermore, zero courses were identified to discuss agroterrorism or agro-crime, bioweapons (including the BTWC and CWC), or intentional contamination, thus highlighting a clear educational gap within these courses. It is also important to note that although courses appear to be up to date with discussions regarding science and technology, it is not known, however, to what extent it is addressed within the courses.

All biosecurity courses were at the Master’s level, exhibiting the requirement for the implementation of biosecurity modules within bachelor’s studies. The target audience for each course varies; however, the typical focus is for veterinarians, farm workers, and governmental staff. The Harper-Adams University (England) offers a Plant Health and Biosecurity course as a PgC, PgD, or MSc and aims its course at government staff with biosecurity responsibilities. This is an important aspect of the course that needs to be mirrored when developing future biosecurity education modules or courses. All courses are delivered via blended learning and offer practical elements.

In total, 103 (52%) of the courses reviewed did not directly address biosecurity, and 54 (27%) courses contained some elements of biosecurity. The lack of implementation of biosecurity within these courses may be accounted for by a number of factors. First, biosecurity within the agribusiness sector is commonly neglected, with the focus usually falling within the topic of public health, dual-use research of concern, and laboratory practise. The lack of visible biosecurity elements does not necessarily mean that these courses do not contain biosecurity-related topics, but only that it is not a visible focus. Second, a lack of detailed course content programmes may also account for the appearance of an absence of biosecurity. For instance, some universities allow access to full course content programmes in comparison to others, which provide summaries and aims of the course. Third, biosecurity is addressed ‘indirectly’ throughout the course, for example, within Veterinary medicine. Only 1 veterinary medicine course (Malta College of Arts, Science and Technology) appeared to directly address biosecurity, though despite this, inevitably, most veterinary courses will address biosecurity or related topics. As such, it may be that biosecurity as a concept is not directly presented; however, biosecurity-related topics are embedded within and throughout the course. Leading from this, courses need to create awareness that they are discussing biosecurity and thus directly name and address the concept.

Additionally, language and definitions of some terminology will play a significant role in addressing the course contents. Some universities provide course content programmes in English; however, some provide translated versions in native languages. This leads to questions regarding how they are translated and the meaning behind the words exchanged. For example, in the English language, there are two separate words for biosecurity and biosafety with two distinct meanings. However, in Portuguese, the concepts of biosecurity and biosafety are the same and are termed *Biossegurança*. As such, it is important to note that these terms may have different meanings in certain languages, academic institutions, or research fields. This leads to further discourse on whether biosecurity is directly addressed, but under different terminology.

It is important to note that limitations of the methodology may account for the ‘absence’ of biosecurity in some courses. For example, the use of EduRank may leave out professionally oriented degrees. As discussed in the above paragraph, veterinary medicine courses will inevitably address biosecurity. It is also important to highlight that this study was limited to publicly available course descriptions. Additionally, the lack of standard classification and course contents for biosecurity highlights that this area needs additional study. However, it is actually showing the clear gap in the area that desperately needs to be improved.

As to the delivery, courses exhibiting clear biosecurity elements appeared to rely on traditional teaching methods, such as lectures with some practical elements, and it is unknown how these courses are evaluated. Without a clear evaluation methodology or data, it is impossible to discuss the impact or potential impact of the course. As such, we recommend alternative methods such as Team-Based Learning (TBL), online courses, case-study simulation activities, and short projects as more effective approaches.

To address this gap, Bachelor’s and Master’s courses related to agribusiness must directly address biosecurity. Life, natural, and social science aspects of the topic must be addressed, including legislation, policy, and relevant conventions (BTWC and CWC). Biosecurity must also be clearly defined to avoid merging with other similar topics (Biosafety). Courses must expand modules to include discussions on agroterrorism, agro-crime, bioweapons, and unintentional/intentional contamination, in addition to the management of a shock event. It is also vital for courses to address the economic, environmental, physical, psychological, and political implications of a shock event and to address the interdisciplinary nature of this topic. Additionally, courses must clearly address advancements made in science and technology. Courses may implement their curriculum via Team-Based Learning, online courses, case-study simulation activities, and short projects. Courses must also have a clearly defined evaluation methodology in order to review the impact and effectiveness of the course. It is also important to highlight that the implementation of biosecurity into these courses should have a target audience of future government or policy staff, veterinarians, farm staff, and any other professional bodies involved in the food supply chain. Thus, the clearly established biosecurity educational gap within the agribusiness sector within universities calls for an internationally coordinated course that contains the aforementioned properties.

## 5 Recommendations and conclusion

Advancements in science and technology, the increasing emergence of zoonotic and plant diseases, the threat of climate change, rising food demand, and the increasingly unstable political conflicts highlight the need to address biosecurity risks to the agribusiness sector within educational institutions.

Within university courses relating to agribusiness, the above results suggest a neglect of focus on biosecurity, including the management and global consequences of a shock event. This neglect is evidenced by only 4% of courses surveyed containing clear biosecurity elements and zero Bachelor’s courses directly addressing Biosecurity. This emphasises the need/requirement to develop a sustainable biosecurity curriculum internationally. Moreover, the curriculum must be devised in a way that can effectively present the natural, life, and social science aspects of the topic with a strong emphasis on legislation, policy, and conventions. The curriculum must also discuss agroterrorism, agro-crime, bioweapons, and accidental/intentional contamination. It is also vital that the curriculum address the economic, environmental, physical, psychological, and political implications of a shock event, including the management of such an event. The curriculum may be implemented and delivered via lectures, Team-Based learning, online courses, case-study simulation activities, and short projects, etc., apart from the traditional class lecturing. The delivery and overall impact of the curriculum must be subsequently evaluated. The most challenging segment of devising a curriculum is how to sustainably implement it. To sustainably implement Biosecurity education into agribusiness-related courses, it is recommended to embed biosecurity into already established courses, building upon concepts already taught to students. Early introduction to these concepts will allow students to utilise these tools throughout academia and into professions. As such, the dramatically low percentage of courses that contain clear biosecurity elements, and the fact that this may indicate a common theme across Europe, leads to calls for an internationally coordinated biosecurity curriculum at universities for the agribusiness sector. The details of designing the course materials, including developing implementation methodologies, and evaluating and assessing, will be addressed in our separate project, in which we will include useful analysis on populations of the countries per biosecurity education course, the regulatory landscape for biosecurity risks in agribusiness, etc. We will also extend this beyond the European countries to include good practises in other countries, such as the United States.

To conclude, biosecurity education with the agribusiness sector at the Bachelor’s and Master’s taught level may be neglected, as evidenced by only 4% of courses directly addressing Biosecurity. There is an urgent need for biosecurity education at Universities within the Agribusiness Sector, and beyond.

## Data Availability

The original contributions presented in the study are included in the article/Supplementary material, further inquiries can be directed to the corresponding author.
