# Supplementary material for: The urgent need for biosecurity education within the agribusiness sector
Source: Front Microbiol. 2026 Feb 11;17:1735642. doi: 10.3389/fmicb.2026.1735642 (PMC12932524; doi:10.3389/fmicb.2026.1735642)
Supplement: Supplementary file 1 [file Data_Sheet_1.docx]

**Appendix List of courses reviewed and the extent of which Biosecurity is addressed.**

| **Country** | **Number** | **University** | **Course level** | **Course name** | **Extent of which Biosecurity is addressed** |
| --- | --- | --- | --- | --- | --- |
| Austria | 1 | University of Natural Resources and Life Sciences, Vienna | Bachelor | Food science and Biotechnology | Unable to access course content.  Module: Bioethics – Vaccines or Weapons (Duel use research of concern)  Full programme content unavailable in English. |
|  | 2 | University of Natural Resources and Life Sciences, Vienna | Bachelor | Agriculture sciences | Unable to access course content.  Safety and health protection in agriculture and forest enterprises: Hazardous substances.  Full programme content unavailable in English. |
|  | 3 | University of Natural Resources and Life Sciences, Vienna | Masters | Food science and technology | Unable to access course content.  Food safety and risk management.  Full programme content unavailable in English. |
|  | 4 | University of Natural Resources and Life Sciences, Vienna | Masters | Environment and Bio-Resource management | Unable to access course content.  Contaminated sites and soil protections, Nuclear safety, Biological monitoring, Biological and Biotechnical plant protection.  Full programme content unavailable in English. |
|  | 5 | University of Natural Resources and Life Sciences, Vienna | Masters | Plant sciences | Unable to access course content.  Agricultural pest diagnostics, Soil-borne pathogens and symbionts.  Full programme content unavailable in English. |
| Belgium | 6 | Ghent University | Masters | Poultry Health sciences | Some Biosecurity elements:  Treatment, Prevention and Diseases of Poultry.   - Course is being phased out. |
|  | 7 | Ghent University | Masters | Sustainable food packaging solutions | No visible Biosecurity elements or intentional contamination. |
| Bulgaria | 8 | Sofia University 'St. Kliment Ohridski' | Bachelor | Ecology and Environmental protection | No visible Biosecurity elements or intentional contamination. |
|  | 9 | Sofia University 'St. Kliment Ohridski' | Bachelor | Biotechnology | No visible Biosecurity elements or intentional contamination. |
|  | 10 | Sofia University 'St. Kliment Ohridski' | Masters | Plant Biotechnology | No visible Biosecurity elements or intentional contamination. |
|  | 11 | Sofia University 'St. Kliment Ohridski' | Masters | Ecological Biotechnology | No visible Biosecurity elements or intentional contamination. |
|  | 12 | Sofia University 'St. Kliment Ohridski' | Masters | Parasitology | Some Biosecurity elements:  Epidemiology  Optional module: Biological control of insects/pests (control of parasites in food products) |
|  | 13 | Sofia University 'St. Kliment Ohridski' | Masters | Plant physiology | No visible Biosecurity elements or intentional contamination. |
|  | 14 | Sofia University 'St. Kliment Ohridski' | Masters | Environmental protection | No visible Biosecurity elements or intentional contamination. |
|  | 15 | Technical University – Sofia | Masters | Food Safety | Some Biosecurity elements:  Food chain legislation and control, Microorganism food safety |
|  | 16 | Agricultural University Plovdiv | Bachelor and Masters | N/A | Course content unavailable in English. |
| Croatia | 17 | University of Zagreb | Bachelor | Agriculture economics | No visible Biosecurity elements or intentional contamination. |
|  | 18 | University of Zagreb | Bachelor | Agricultural engineering | No visible Biosecurity elements or intentional contamination. |
|  | **19** | **University of Zagreb** | **Masters** | **Farm Biosecurity** | **Clear Biosecurity elements (See Table 3).** |
|  | 20 | University of Zagreb | Masters | Forensic Veterinary Medicine | Some Biosecurity elements:  Epidemiology, identification of criminal acts. |
|  | **21** | **University of Zagreb** | **Masters** | **Agri-Food Chain Microbiology** | **Clear Biosecurity elements (See Table 3).** |
|  | 22 | University of Zagreb | Masters | Veterinary epidemiology | Some Biosecurity elements:  Epidemiology, control and management of Infectious diseases. |
|  | 23 | JosipJuraj Strossmayer University of Osijek | Bachelor | Plant production | Unable to access course content. |
|  | 24 | JosipJuraj Strossmayer University of Osijek | Bachelor | Agro-Economics | Unable to access course content. |
|  | 25 | JosipJuraj Strossmayer University of Osijek | Masters | Plant production | Unable to access course content. |
|  | 26 | JosipJuraj Strossmayer University of Osijek | Masters | Organic agriculture | Unable to access course content. |
| Republic of Cyprus | 27 | Cyprus University of Technology | Bachelor | Agriculture sciences, Biotechnology and Food Science – Plant production science and technology | Some Biosecurity elements:  Crop production, plant protection, Plant breeding and genetics. |
|  | 28 | Cyprus University of Technology | Bachelor | Agriculture sciences, Biotechnology and Food Science – Food science and technology | Some Biosecurity elements:  Food quality and safety, Food Science and Technology of products derived from Plants and Animals. |
|  | 29 | Cyprus University of Technology | Bachelor | Agriculture sciences, Biotechnology and Food Science -Animal husbandry and dairying | Some Biosecurity elements:  Animal breeding and genetics, Zoonoses, Food crisis, Feed Science and Technology. |
|  | 30 | Cyprus University of Technology | Masters | Agriculture Biotechnology | Some Biosecurity elements:  Bioethics and Biosafety, Genetic engineering |
|  | 31 | University of Cyprus | Masters | Biodiversity and Ecology | No visible Biosecurity elements or intentional contamination. |
| Czech Republic | 32 | Czech University of Life Sciences | Bachelor | Agriculture and Food | Unable to access course content. |
|  | 33 | Czech University of Life Sciences | Masters | Sustainable agriculture and food security | Unable to access course content. |
|  | 34 | Charles University | Bachelor | Environmental protection | Unable to access course content. |
|  | 35 | Charles University | Bachelor | Ecology and Evolutionary biology | Unable to access course content. |
|  | 36 | Charles University | Masters | Ecology | No visible Biosecurity elements or intentional contamination. |
|  | 37 | Charles University | Masters | Experimental Plant Biology | No visible Biosecurity elements or intentional contamination. |
|  | 38 | Charles University | Masters | Parasitology and infection biology | Some Biosecurity elements:  Control of pathogens, epidemiology |
| Denmark | 39 | DTU National Food Institute | Masters | Sustainable and safe food production | Some Biosecurity elements:  Epidemiology of food borne diseases. |
|  | 40 | University of Copenhagen | Masters | Agriculture | Some Biosecurity elements:  Epidemiology of plant diseases. |
|  | 41 | University of Copenhagen | Masters | Food science and technology | No visible Biosecurity elements or intentional contamination. |
| England | **42** | **Harper-Adams University** | **Masters** | **Plant Health and Biosecurity** | **Clear Biosecurity elements (See Table 3).** |
|  | 43 | Newcastle University | Bachelor | Agriculture | Some Biosecurity elements:  Control of crop pests and diseases in the food-supply chain. |
|  | 44 | Newcastle University | Bachelor | Agriculture with farm business management | Some Biosecurity elements:  Control of crop pests and diseases in the food-supply chain. |
|  | 45 | Newcastle University | Bachelor | Animal Science | No visible Biosecurity elements or intentional contamination. |
|  | 46 | Newcastle University | Bachelor | Agri-Business management | No visible Biosecurity elements or intentional contamination. |
|  | 47 | Newcastle University | Masters | Agriculture and Environmental science | No visible Biosecurity elements or intentional contamination. |
|  | 48 | Royal Agriculture University | Bachelor | Agriculture | No visible Biosecurity elements or intentional contamination. |
|  | 49 | Royal Agriculture University | Masters | Sustainable agriculture and food security | No visible Biosecurity elements or intentional contamination. |
|  | 50 | Royal Agriculture University | Masters | Agricultural technology and innovation | No visible Biosecurity elements or intentional contamination. |
|  | 51 | Lancaster University | Masters | Environmental management | No visible Biosecurity elements or intentional contamination. |
|  | 52 | Lancaster University | Masters | Political Ecology | No visible Biosecurity elements or intentional contamination. |
|  | 53 | Lancaster University | Masters | Global Food Security | No visible Biosecurity elements or intentional contamination. |
|  | 54 | Royal Veterinary Collage | Bachelor | BioVeterinary sciences | Some Biosecurity elements:  Epidemiology of pests and tropical diseases. |
|  | 55 | Royal Veterinary Collage | Bachelor | Biological Sciences (Wildlife sciences) | Some Biosecurity elements:  Epidemiology of wildlife diseases. |
|  | 56 | Royal Veterinary Collage | Bachelor | Animal biology, Behaviour, Welfare and Ethics | No visible Biosecurity elements or intentional contamination. |
| Estonia | 57 | University of Tartu | Bachelor | Protection of Biology | No visible Biosecurity elements or intentional contamination. |
|  | 58 | University of Tartu | Masters | Biology and Eco-innovation | No visible Biosecurity elements or intentional contamination. |
|  | 59 | Estonian University of Life Sciences | Bachelor | Veterinary medicine | Some Biosecurity elements:  Relationship between Animal and Human health. |
| Finland | 60 | University of Helsinki | Bachelor | Agriculture science | Unable to access course content.  Full programme content unavailable in English. |
|  | 61 | University of Helsinki | Bachelor | Environmental and Food economy | Unable to access course content.  Full programme content unavailable in English. |
|  | 62 | University of Helsinki | Masters | Agricultural sciences | No visible Biosecurity elements or intentional contamination. |
|  | 63 | University of Turku | Masters | Food development | No visible Biosecurity elements or intentional contamination. |
| France | 64 | University of Montpellier | Bachelor | Biological engineering | No visible Biosecurity elements or intentional contamination. |
|  | 65 | University of Montpellier | Masters | Biology – AgroSciences | No visible Biosecurity elements or intentional contamination. |
|  | 66 | University of Montpellier | Master | Nutrition and Food science | No visible Biosecurity elements or intentional contamination. |
|  | 67 | Institut Polytechnique de Paris | Bachelor | Science | No visible Biosecurity elements or intentional contamination. |
|  | 68 | Institut Polytechnique de Paris | Masters | Biology and Health | No visible Biosecurity elements or intentional contamination. |
| Germany | 69 | University of Hohenheim | Masters | Agricultural sciences | No visible Biosecurity elements or intentional contamination. |
|  | 70 | University of Hohenheim | Masters | Advisory and Innovation Servies in Agri-food systems | No visible Biosecurity elements or intentional contamination. |
|  | 71 | University of Hohenheim | Masters | Animal sciences | No visible Biosecurity elements or intentional contamination. |
|  | 72 | University of Gottingen | Bachelor | Agricultural sciences | No visible Biosecurity elements or intentional contamination. |
|  | 73 | University of Gottingen | Masters | Agricultural sciences | No visible Biosecurity elements or intentional contamination. |
|  | 74 | University of Gottingen | Masters | Crop protection | No visible Biosecurity elements or intentional contamination. |
|  | 75 | University of Gottingen | Masters | Agribusiness | No visible Biosecurity elements or intentional contamination. |
| Greece | 76 | Aristotle University of Thessaloniki | Bachelor | Agriculture | Some Biosecurity elements:  Plant and crop protection. |
|  | 77 | Aristotle University of Thessaloniki | Bachelor | Veterinary medicine | Some Biosecurity elements:  Pathology and Epidemiology. |
|  | 78 | Aristotle University of Thessaloniki | Masters | Sustainable agricultural production and climate change | Unable to access course content.  Full programme content unavailable in English. |
|  | 79 | Agricultural University of Athens | Bachelor | Crop science | Some Biosecurity elements:  Crop protection and control of pests and diseases. |
|  | 80 | Agricultural University of Athens | Masters | Crop science | Some Biosecurity elements:  Crop protection and control of pests and diseases. |
|  | 81 | Agricultural University of Athens | Masters | Food and Agribusiness | No visible Biosecurity elements or intentional contamination. |
| Hungary | 82 | University of Debrecen | Bachelor | Agricultural engineering | No visible Biosecurity elements or intentional contamination. |
|  | 83 | University of Debrecen | Bachelor | Food engineering | No visible Biosecurity elements or intentional contamination. |
|  | 84 | University of Debrecen | Masters | Crop production engineering | Some Biosecurity elements:  Control of pests and diseases. |
|  | 85 | University of Debrecen | Masters | Food safety and quality engineering | Some Biosecurity elements:  Foodborne diseases |
|  | 86 | University of Debrecen | Masters | Plant protection | Some Biosecurity elements:  Control of pests and pathogens. |
|  | 87 | Hungarian University of Agriculture and Life sciences | Bachelor | Food engineering | No visible Biosecurity elements or intentional contamination. |
|  | 88 | Hungarian University of Agriculture and Life sciences | Masters | Agricultural Biotechnology | No visible Biosecurity elements or intentional contamination. |
|  | 89 | Hungarian University of Agriculture and Life sciences | Masters | Food engineering | No visible Biosecurity elements or intentional contamination. |
|  | 90 | Hungarian University of Agriculture and Life sciences | Masters | Crop production engineering | Some Biosecurity elements:  Control of pests |
|  | 91 | Hungarian University of Agriculture and Life sciences | Bachelor | Wildlife engineering | No visible Biosecurity elements or intentional contamination. |
|  | 92 | Hungarian University of Agriculture and Life sciences | Masters | Wildlife engineering | No visible Biosecurity elements or intentional contamination. |
| Iceland | 93 | Agricultural University of Iceland | Bachelor | Agriculture, food and the environment | Some Biosecurity elements:  Livestock biology and control of pathogens. |
|  | 94 | Agricultural University of Iceland | Masters | Agricultural studies | No visible Biosecurity elements or intentional contamination. |
| Ireland | 95 | University College Dublin | Bachelor | Agricultural sciences | No visible Biosecurity elements or intentional contamination. |
|  | 96 | University College Dublin | Bachelor | Animal sciences | Some Biosecurity elements:  Control of Zoonotic diseases. |
|  | 97 | University College Dublin | Bachelor | Crop protection | Some Biosecurity elements:  Control of plant disease and pests. |
|  | 98 | University College Dublin | Bachelor | Food science | No visible Biosecurity elements or intentional contamination. |
|  | 99 | University College Dublin | Masters | Food regulatory affairs | Some Biosecurity elements:  Linked to Veterinary Public Health. Relationship from Farm to Table. |
|  | 100 | University College Dublin | Masters | Food safety | Some Biosecurity elements:  Risk assessments and Biological hazards. |
|  | 101 | University College Cork | Bachelor | Agriculture sciences | No visible Biosecurity elements or intentional contamination. |
|  | 102 | University College Cork | Bachelor | Applied Plant biology | No visible Biosecurity elements or intentional contamination. |
|  | 103 | University College Cork | Masters | Animal behaviour and welfare | No visible Biosecurity elements or intentional contamination. |
|  | 104 | University College Cork | Masters | Food science | No visible Biosecurity elements or intentional contamination. |
|  | 105 | University College Cork | Masters | Plant Biology | Some Biosecurity elements: Crop protection and consequences of Climate Change. |
| Italy | 106 | University of Bologna | Bachelor | Agricultural technology | No visible Biosecurity elements or intentional contamination. |
|  | 107 | University of Bologna | Bachelor | Animal production | Some Biosecurity elements:  Epidemiology, Parasitology and Veterinary Pathology |
|  | 108 | University of Bologna | Bachelor | Food technology and nutrition | Some Biosecurity elements:  Hygiene and inspection of food animal origin, disease and pests for food crops. |
|  | 109 | University of Bologna | Masters | Agricultural science and technology | Some Biosecurity elements:  Crop protection and pest control. |
|  | **110** | **University of Bologna** | **Masters** | **Food safety and Food risk management** | **Clear Biosecurity elements (See Table 3).** |
|  | 111 | University of Milan | Bachelor | Agrifood Biotechnology | No visible Biosecurity elements or intentional contamination. |
|  | 112 | University of Milan | Bachelor | Science and technology for sustainable food | No visible Biosecurity elements or intentional contamination. |
|  | 113 | University of Milan | Masters | Crop and plant sciences | No visible Biosecurity elements or intentional contamination. |
| Latvia | 114 | Latvia University of Life Sciences and Technologies | Bachelor | Sustainable agriculture | No visible Biosecurity elements or intentional contamination. |
|  | 115 | Latvia University of Life Sciences and Technologies | Masters | Food science | No visible Biosecurity elements or intentional contamination. |
|  | 116 | University of Latvia | Bachelor | Biotechnology and Bioengineering | No visible Biosecurity elements or intentional contamination. |
| Liechtenstein | 117 | University of Liechtenstein | N/A | N/A | Unable to access relevant Agribusiness courses. |
|  | 118 | International Academy for Philosophy | N/A | N/A | Unable to access relevant Agribusiness courses. |
|  | 119 | Liechtenstein institute | N/A | N/A | Unable to access relevant Agribusiness courses. |
|  | 120 | Private University in the Principality of Liechtenstein | N/A | N/A | Unable to access relevant Agribusiness courses. |
| Lithuania | 121 | Vytautas Magnus University | Bachelor | Biotechnology | No visible Biosecurity elements or intentional contamination. |
|  | 122 | Vytautas Magnus University | Bachelor | Environmental science and protection | No visible Biosecurity elements or intentional contamination. |
|  | 123 | Vytautas Magnus University | Masters | Ecology and Climate change | No visible Biosecurity elements or intentional contamination. |
|  | 124 | Vytautas Magnus University | Masters | Agronomy | Some Biosecurity elements:  Pest control |
|  | 125 | Kaunas University of Technology | Bachelor | Food science and technology | No visible Biosecurity elements or intentional contamination. |
|  | 126 | Kaunas University of Technology | Bachelor | Industrial Biotechnology | No visible Biosecurity elements or intentional contamination. |
|  | 127 | Kaunas University of Technology | Masters | Food science and nutrition | No visible Biosecurity elements or intentional contamination. |
|  | 128 | Kaunas University of Technology | Masters | Food technology and innovation | No visible Biosecurity elements or intentional contamination. |
|  | 129 | Kaunas University of Technology | Masters | Industrial Biotechnology | No visible Biosecurity elements or intentional contamination. |
| Luxembourg | 130 | University of Luxembourg | N/A | N/A | Unable to access relevant Agribusiness courses. |
|  | 131 | Sacred Hearts university | N/A | N/A | Unable to access relevant Agribusiness courses. |
| Malta | 132 | L-Universitά tά Malta | Bachelor | Rural science and food systems | No visible Biosecurity elements or intentional contamination. |
|  | 133 | L-Universitά tά Malta | Masters | Rural science and food systems | No visible Biosecurity elements or intentional contamination. |
|  | 134 | Malta college of Arts, Science and Technology | Bachelor | Animal management and veterinary nursing | No visible Biosecurity elements or intentional contamination. |
|  | 135 | Malta college of Arts, Science and Technology | Bachelor | Environmental health | No visible Biosecurity elements or intentional contamination. |
|  | 136 | Malta college of Arts, Science and Technology | Bachelor | Environmental science and sustainable technology | No visible Biosecurity elements or intentional contamination. |
|  | 137 | Malta college of Arts, Science and Technology | Masters | Environmental science | Some Biosecurity elements:  Natural hazards, relationship between Agriculture and conflicts. |
|  | **138** | **Malta college of Arts, Science and Technology** | **Masters** | **Veterinary science** | **Clear Biosecurity elements (See Table 3).** |
| The Netherlands | 139 | Wageningen University | Bachelor | Food technology | No visible Biosecurity elements or intentional contamination. |
|  | 140 | Wageningen University | Bachelor | Animal sciences | No visible Biosecurity elements or intentional contamination. |
| Norway | 141 | Norwegian University of Life sciences | Masters | Animal sciences | No visible Biosecurity elements or intentional contamination. |
|  | 142 | Norwegian University of Life sciences | Masters | Plant sciences | No visible Biosecurity elements or intentional contamination. |
| Poland | 143 | University of Agriculture in Krakow | Bachelor | Food technology and human nutrition | No visible Biosecurity elements or intentional contamination. |
|  | 144 | University of Agriculture in Krakow | Bachelor | Food processing, safety and quality | No visible Biosecurity elements or intentional contamination. |
| Portugal | 145 | Instituto Superior D agronomia Universidade de Lisboa | Bachelor | Food science and engineering | Some Biosecurity elements: Biochemical, physicochemical and microbiological modifications to food. |
|  | 146 | Instituto Superior D agronomia Universidade de Lisboa | Bachelor | Animal production engineering | Some Biosecurity elements:  Control of pathogens |
|  | 147 | Instituto Superior D agronomia Universidade de Lisboa | Masters | Food science and engineering | Some Biosecurity elements: Biochemical, physicochemical and microbiological modifications to food. |
|  | 148 | Instituto Superior D agronomia Universidade de Lisboa | Masters | Animal production engineering | Some Biosecurity elements:  Control of pathogens |
| Romania | 149 | University of Agricultural Sciences and Veterinary Medicine of Cluj-Napoca | Bachelor | Agriculture | Unable to access course content.  Full programme content unavailable in English. |
|  | 150 | University of Agricultural Sciences and Veterinary Medicine of Cluj-Napoca | Bachelor | Veterinary medicine | Unable to access course content.  Full programme content unavailable in English. |
|  | 151 | University of Agricultural Sciences and Veterinary Medicine of Cluj-Napoca | Masters | Agriculture | Unable to access course content.  Full programme content unavailable in English. |
|  | 152 | University of Agronomic sciences and veterinary medicine of Bucharest | Bachelor | Agriculurre | No visible Biosecurity elements or intentional contamination. |
|  | 153 | University of Agronomic sciences and veterinary medicine of Bucharest | Bachelor | Animal science | No visible Biosecurity elements or intentional contamination. |
|  | 154 | University of Agronomic sciences and veterinary medicine of Bucharest | Bachelor | Veterinary medicine | Some Biosecurity elements:  Infectious diseases, pests and veterinary pathology |
|  | **155** | **University of Agronomic sciences and veterinary medicine of Bucharest** | **Masters** | **Food biosafety** | **Clear Biosecurity elements (See Table 3).** |
|  | **156** | **University of Agronomic sciences and veterinary medicine of Bucharest** | **Masters** | **Food safety and Biosecurity** | **Clear Biosecurity elements (See Table 3).** |
| Scotland | 157 | University of Edinburgh | Bachelor | Ecological and Environmental science | No visible Biosecurity elements or intentional contamination. |
|  | 158 | University of Edinburgh | Bachelor | Veterinary medicine | No visible Biosecurity elements or intentional contamination. |
|  | 159 | University of Edinburgh | Masters | Animal behaviour and welfare | No visible Biosecurity elements or intentional contamination. |
|  | 160 | University of Edinburgh | Masters | Biodiversity and Taxonomy of Plants | No visible Biosecurity elements or intentional contamination. |
|  | 161 | University of Edinburgh | Masters | Food security | Some Biosecurity elements:  Role of genetics and orphan crops |
| Slovakia | 162 | The Slovak University of Agriculture in Nitra | Bachelor | Agrobiotechnology | Unable to access course content. |
|  | 163 | The Slovak University of Agriculture in Nitra | Bachelor | Agri-food | Unable to access course content. |
|  | 164 | The Slovak University of Agriculture in Nitra | Bachelor | Food safety and control | Unable to access course content. |
|  | 165 | The Slovak University of Agriculture in Nitra | Bachelor | Livestock production management | Unable to access course content. |
|  | 166 | The Slovak University of Agriculture in Nitra | Masters | Agrobiotechnology | Unable to access course content. |
|  | 167 | The Slovak University of Agriculture in Nitra | Masters | Food safety and control | Unable to access course content. |
|  | 168 | The Slovak University of Agriculture in Nitra | Masters | Genetic technologies in Agrobiology | Unable to access course content. |
|  | 169 | The Slovak University of Agriculture in Nitra | Masters | Crop production management | Unable to access course content. |
|  | 170 | The Slovak University of Agriculture in Nitra | Masters | Food technology | Unable to access course content. |
|  | 171 | Comenius University Bratislava | Bachelor | Environmental studies | No visible Biosecurity elements or intentional contamination. |
|  | 172 | Comenius University Bratislava | Masters | Environmental studies | No visible Biosecurity elements or intentional contamination. |
| Slovenia | 173 | University of Maribor | Bachelor | Agriculture and environment | No visible Biosecurity elements or intentional contamination. |
|  | 174 | University of Maribor | Bachelor | Agronomy | No visible Biosecurity elements or intentional contamination. |
|  | 175 | University of Maribor | Bachelor | Animal science | No visible Biosecurity elements or intentional contamination. |
|  | 176 | University of Maribor | Masters | Agriculture | No visible Biosecurity elements or intentional contamination. |
|  | 177 | University of Maribor | Masters | Food safety in the Agri-food supply chain | Some Biosecurity elements:  Biological risks and risk assessments. |
|  | 178 | University of Ljubl jana | Bachelor | Agriculture – Animal production | Some Biosecurity elements:  Crop protection |
|  | 179 | University of Ljubl jana | Bachelor | Food science and nutrition | Some Biosecurity elements:  Epidemiology |
|  | 180 | University of Ljubl jana | Bachelor | Agriculture – Agronomy | No visible Biosecurity elements or intentional contamination. |
|  | 181 | University of Ljubl jana | Bachelor | Biotechnology | Some Biosecurity elements:  Biosafety and regulation in Biotechnology, |
|  | 182 | University of Ljubl jana | Masters | Agronomy | Some Biosecurity elements:  Biological control, control of pests and diseases. |
|  | 183 | University of Ljubl jana | Masters | Biotechnology | No visible Biosecurity elements or intentional contamination. |
|  | 184 | University of Ljubl jana | Masters | Food science | No visible Biosecurity elements or intentional contamination. |
|  | 185 | University of Ljubl jana | Masters | Animal science | Some Biosecurity elements:  Food safety – Farm to Fork |
|  | 186 | University of Ljubl jana | Masters | Ecology and Biodiversity | No visible Biosecurity elements or intentional contamination. |
| Spain | 187 | Universitat de Lleida School of Agrifood and Forestry Engineering and Veterinary medicine | Bachelor | Food science and technology | No visible Biosecurity elements or intentional contamination. |
|  | 188 | Universitat de Lleida School of Agrifood and Forestry Engineering and Veterinary medicine | Bachelor | Agricultural and food engineering | No visible Biosecurity elements or intentional contamination. |
|  | 189 | Universitat de Lleida School of Agrifood and Forestry Engineering and Veterinary medicine | Double Bachelor | Veterinary medicine, animal science + production | No visible Biosecurity elements or intentional contamination. |
|  | 190 | Universitat de Lleida School of Agrifood and Forestry Engineering and Veterinary medicine | Masters | Swine health and production | No visible Biosecurity elements or intentional contamination. |
|  | 191 | Universitat de Lleida School + University of Zaragoza Complutense University of Madrid | Masters | Integrated crop production | Some Biosecurity element:  Detect and diagnose crop disease, pests and weeds. |
|  | 192 | Universitat de Lleida School of Agrifood and Forestry Engineering and Veterinary medicine | Masters | Agricultural engineering | No visible Biosecurity elements or intentional contamination. |
| Sweden | 193 | University of Agricultural sciences | Bachelor | Animal Agronomist programme | No visible Biosecurity elements or intentional contamination. |
|  | 194 | University of Agricultural sciences | Bachelor | Food Agronomist programme | Some Biosecurity elements:  Risk assessments |
|  | 195 | University of Agricultural sciences | Bachelor | Agronomist programme crop and plants | Some Biosecurity elements:  Control of diseases and pests. |
|  | 196 | University of Agricultural sciences | Bachelor | Food programme | No visible Biosecurity elements or intentional contamination. |
|  | 197 | University of Agricultural sciences | Bachelor | Crop production programme | No visible Biosecurity elements or intentional contamination. |
|  | 198 | University of Agricultural sciences | Masters | Agricultural, food and environmental policy | No visible Biosecurity elements or intentional contamination. |
|  | 199 | University of Agricultural sciences | Masters | Animal Agronomist programme | No visible Biosecurity elements or intentional contamination. |
|  | 200 | University of Agricultural sciences | Masters | Food Agronomist programme | Some Biosecurity elements:  Risk assessments |
|  | 201 | University of Agricultural sciences | Masters | Agronomist programme crop and plants | Some Biosecurity elements:  Control of diseases and pests. |
|  | 202 | University of Agricultural sciences | Masters | Animal science | No visible Biosecurity elements or intentional contamination. |
|  | 203 | University of Agricultural sciences | Masters | Food control | No visible Biosecurity elements or intentional contamination. |
|  | 204 | University of Agricultural sciences | Masters | Plant biology for sustainable production | Some Biosecurity elements:  Crop protection from diseases. |
|  | 205 | University of Agricultural sciences | Masters | Sustainable food systems | No visible Biosecurity elements or intentional contamination. |
